# Functional Convergence of Neurons Generated in the Developing and Adult Hippocampus

**DOI:** 10.1371/journal.pbio.0040409

**Published:** 2006-11-21

**Authors:** Diego A Laplagne, M. Soledad Espósito, Verónica C Piatti, Nicolás A Morgenstern, Chunmei Zhao, Henriette van Praag, Fred H Gage, Alejandro F Schinder

**Affiliations:** 1 Fundación Instituto Leloir, Buenos Aires, Argentina; 2 Laboratory of Genetics, The Salk Institute for Biological Studies, La Jolla, California, United States of America; Massachusetts General Hospital, United States of America

## Abstract

The dentate gyrus of the hippocampus contains neural progenitor cells (NPCs) that generate neurons throughout life. Developing neurons of the adult hippocampus have been described in depth. However, little is known about their functional properties as they become fully mature dentate granule cells (DGCs). To compare mature DGCs generated during development and adulthood, NPCs were labeled at both time points using retroviruses expressing different fluorescent proteins. Sequential electrophysiological recordings from neighboring neurons of different ages were carried out to quantitatively study their major synaptic inputs: excitatory projections from the entorhinal cortex and inhibitory afferents from local interneurons. Our results show that DGCs generated in the developing and adult hippocampus display a remarkably similar afferent connectivity with regard to both glutamate and GABA, the major neurotransmitters. We also demonstrate that adult-born neurons can fire action potentials in response to an excitatory drive, exhibiting a firing behavior comparable to that of neurons generated during development. We propose that neurons born in the developing and adult hippocampus constitute a functionally homogeneous neuronal population. These observations are critical to understanding the role of adult neurogenesis in hippocampal function.

## Abstract 2

Adult neurogenesis in the hippocampus generates neurons with striking functional similarity to neurons born during development, indicating that adult-born neurons incorporate normally into hippocampal circuits.

## Introduction

The dentate gyrus (DG) of the adult hippocampus contains neural progenitor cells (NPCs) that divide, differentiate, and migrate to produce functional neurons that become incorporated into the existing hippocampal circuitry [1–8]. Increasing evidence points to the existence of a complex bidirectional interaction between adult neurogenesis and hippocampal function. Manipulations affecting adult neurogenesis can alter some forms of hippocampus-dependent learning and behavior. In turn, the rate of neurogenesis can be regulated by the environment and by hippocampus-dependent learning tasks [9–15]. In pathological states of the brain such as epilepsy, the rate of adult neurogenesis can be significantly increased, and newly generated neurons can migrate to abnormal locations, become engaged in synchronous neuronal activity, and develop aberrant connections [16–19].

The contribution of adult-born neurons to the healthy hippocampus may reside in the renewal and replacement of dying neurons within a single functional population. Alternatively, adult neurogenesis could result in the continuous incorporation of neurons with equivalent functional properties, or neurons with properties that differ from those of dentate granule cells (DGCs) generated in the developing hippocampus [1,3,5,10,20–22]. These putative distinct properties of adult-born DGCs (i.e., different connectivity or plasticity) could be expressed during their early development and/or after reaching maturity. Adult-born DGCs need to establish appropriate connections in an environment that is highly complex and largely different from that of a developing brain. The perinatal hippocampus undergoes massive neuroblast migration and extensive neuronal wiring (networks are beginning to assemble), and its activity is dominated by GABAergic interneurons exerting depolarizing actions [23–25]. In contrast, the adult hippocampus is a mature substrate where glutamatergic and GABAergic networks are fully established and fewer developing neurons undergo radial migration toward the molecular layer. Despite these remarkable differences, we and others have recently demonstrated that the initial sequence of afferent connectivity of DGCs maturing in the adult hippocampus recapitulates the steps followed during development: they become excitable, then receive GABAergic afferents and only then glutamatergic inputs [26–33]. While developing, however, adult-born DGCs exhibit functional properties that are different from those of fully mature neurons [22,28,29,31]. Whether they remain as a distinct functional population upon reaching maturity remains unknown.

DGCs are the principal neurons of the DG, which constitutes the main gateway to the hippocampus [34]. The relevance of DGCs to the hippocampal network is largely determined by the timing, balance, and location of glutamatergic and GABAergic inputs. The major excitatory afferents impinging onto DGCs arise from stellate neurons of the medial and lateral entorhinal cortex (EC), projecting their axons through the medial perforant path (MPP) and lateral perforant path (LPP), respectively [34]. These two pathways convey distinct information to the dorsal hippocampus, as medial, but not lateral, EC is relevant for encoding spatial information [35]. Inhibitory inputs onto DGCs arise from a diversity of hippocampal GABAergic interneurons [36]. Different classes of interneurons innervate the somatic or dendritic domains of target neurons, and it is commonly thought that dendritic afferents modulate the excitatory input onto principal neurons, whereas perisomatic afferents exert a tight control of their output and firing synchrony [34,36–38].

In a previous study, we have shown that neurons generated in the adult DG exhibit intrinsic membrane properties and overall morphological features that are typical of DGCs [1]. Their functional integration in the hippocampal network was demonstrated by the presence of spontaneous and evoked postsynaptic currents [1]. However, the extent to which fully mature neurons generated during development and adulthood become functionally equivalent is critical to understanding their role in hippocampal function, and it has remained unaddressed. In the present study, we have retrovirally labeled neurons generated in the developing and adult DG to compare their integration into the excitatory and inhibitory networks. Our data reveal that neurons generated at these distinct stages present a remarkably similar afferent connectivity. The similarity is also evidenced by the firing behavior elicited by stimulation of excitatory entorhinal afferents.

## Results

### Fluorescent Labeling of DGCs Generated in the Developing and Adult Hippocampus

Due to their continuous generation, DGCs of all ages are present at any given time point in the adult DG. Consequently, unambiguous identification of mature DGCs born in the developing versus the adult hippocampus in brain slices has been the main obstacle to comparing their characteristics. To overcome this problem, we have used a retroviral expression strategy [1,28,39] to label dividing NPCs of the developing (postnatal day 7; P7) and adult (P42) hippocampus using fluorescent proteins (Figure 1A and 1B). This strategy has allowed us to label neurons generated at different developmental stages of the same mice using green and red fluorescent proteins (GFP and RFP, respectively). Thus, DGCs derived from NPCs labeled in P7 pups express GFP (GFP^+^), and those derived from NPCs labeled in adult mice express RFP (RFP^+^), making it possible to distinguish those neurons in brain slices obtained from older mice (Figure 1C and 1D).

**Figure 1:**
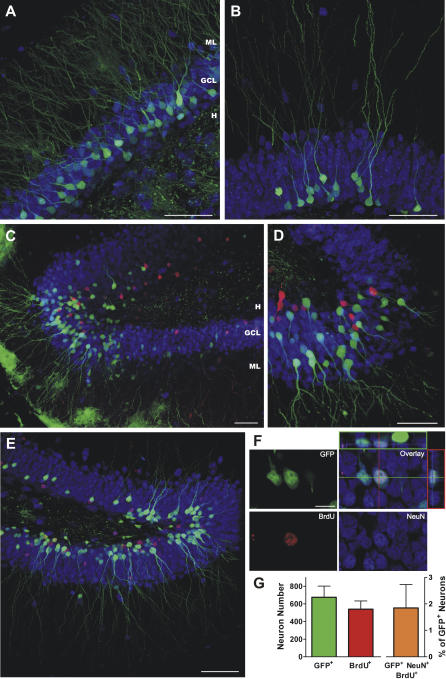
Fluorescent Labeling of DGCs Born during Early Postnatal and Adult Neurogenesis (A) and (B) Retroviral delivery of GFP into DGCs generated at P7 (A) and P42 (B), analyzed 7 wk after each injection. The GCL was labeled by immunohistochemistry for the neuronal marker NeuN (blue). Images are merges of 27 (A) and 21 (B) confocal planes taken from coronal sections (40-μm thick). H, hilus; ML, molecular layer. (C) and (D) Double retroviral labeling of DGCs generated at P7 (GFP^+^, green) and P42 (RFP^+^, red). Images are merges of nine (C) and 20 (D) confocal planes taken from fixed transverse sections of the DG (400-μm thick) from 13-wk-old mice. (E) Double labeling of DGCs with GFP (green) and BrdU (red): intrahippocampal injections of CAG-GFP retrovirus in P7 were followed by daily injections of BrdU carried out from P21 to P25; brains were analyzed at P53. The image is a merge of 16 confocal planes. (F) Example of a sporadic event of co-localization of GFP, BrdU, and NeuN shown by a single optical section for the green, red, and blue channels. Their overlay is shown together with the orthogonal projections onto the *x-z* (top) and *y-z* (right) planes. (G) Number of GFP^+^ or BrdU^+^ cells per mouse (left) and the percentage of GFP^+^ cells showing BrdU label (right). Data are mean ± standard error of the mean (SEM) (*n* = 3 mice). Scale bars indicate 50 μm (A–E) or 10 μm (F).

If P7-labeled NPCs were to continue to proliferate, the ages of GFP^+^ neurons might be highly heterogeneous. We have therefore quantified this phenomenon by injecting bromodeoxyuridine (BrdU) into P21–P25 mice (five daily injections) carrying GFP^+^ cells labeled at P7. Co-localization of GFP, BrdU, and the neuronal marker NeuN was assessed 4 wk later (Figure 1E–1G), at which time GFP^+^ neurons expressing BrdU should exhibit a mature morphology [28]. Indeed, the low incidence (<2%) of BrdU labeling in GFP^+^ neurons and the mature morphology observed in GFP^+^ neurons indicate that most NPCs labeled at P7 have stopped dividing before the onset of BrdU injections, validating double retroviral infections as a reliable method to identify in the adult hippocampus neurons born at different times (Figure 1G).

### Similarity of Entorhinal Afferents of DGCs Born in Pups and Adult Mice

The main excitatory afferents to DGCs arise from the EC through the medial and lateral perforant paths, which are known to underlie distinct hippocampal processing and to exhibit distinct physiological properties [35,40,41]. Comparison of evoked excitatory postsynaptic currents (EPSCs) was done in paired experiments in which a GFP^+^ (P7; pup-born) and a neighboring RFP^+^ (P42; adult-born) neuron were recorded successively, while maintaining the position and strength of the extracellular stimulus. The ages of neurons at the time of recording were 18 wk (P7) and approximately 13 wk (P42) (Figure 2A–2C; see Materials and Methods). EPSCs were elicited by perforant-path stimulation in acute hippocampal slices obtained 13 wk after adult injection in the presence of the GABA_A_ receptor antagonist bicuculline methiodide (BMI; 20 μM). EPSCs were fully blocked by DNQX (6, 7-dinitroquinoxaline-2, 3-dione; 20 μM) and AP-5 (2-amino-5-phosphonopentanoic acid; 100 μM) (Figure 2D). Appropriate separation of medial and lateral stimulation was corroborated in GFP^+^ cells by the facilitation elicited by paired-pulse stimulation of the LPP, the depression induced by paired-pulse stimulation of the MPP [41], and by the slower rise time of LPP-elicited EPSCs [40] (Figure 2E and 2F). Separation of MPP and LPP was equally evident in RFP^+^ neurons.

**Figure 2:**
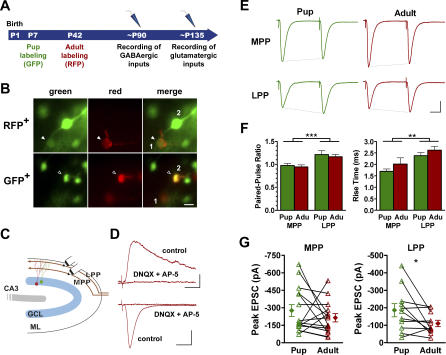
Entorhinal Glutamatergic Afferents onto Mature Neurons Generated in the Developing and Adult Hippocampus (A) Schematic diagram of experimental design for retroviral labeling and electrophysiological recordings. (B) A paired experiment. Upper row: a RFP^+^ neuron was patched (cell “1”) and filled with Alexa Fluor 488 (green). Lower row: a neighboring GFP^+^ neuron (cell “2”) was subsequently patched and filled with Alexa Fluor 594 (red). Both DGCs are in the same field, although at different focal planes (see merge channel). At the end of the experiment, both cells display green and red fluorescence (merge). Scale bar indicates 10 μm. (C) Schematic diagram of the DG showing the position of the bipolar electrodes for the stimulation of the MPP and LPP. (D) Example of EPSCs from an adult-born DGC at V_hold_ = +50 mV (top) and −80 mV (bottom) elicited by LPP stimulation recorded in the presence of vehicle (“control”) or 20 μM DNQX + 100 μM AP-5. Scale bars indicate 20 ms, 40 pA (top) or 20 pA (bottom). Similar properties were observed in DGCs born during development (unpublished data) (E) Example of a paired experiment. Sequential recordings of two neighboring pup and adult DGCs in response to paired-pulse stimulation (100-ms interval) of the MPP and LPP. Scale bars represent 25 ms, 50 pA. (F) Paired-pulse ratio (left) and 20%–80% rise time (right) of EPSCs recorded from pup and adult DGCs upon stimulation of MPP and LPP (*n* = 11 to 15) Paired-pulse ratio was measured as the ratio between the amplitude of the second pulse over the first. Two-way analysis of variance (ANOVA) revealed a significant effect of MPP versus LPP for paired-pulse ratio (*p* = 0.0002) and rise time (*p* = 0.0017), but no significant effect of pup versus adult (Adu) for either parameter. (G) Peak EPSC amplitude recorded in paired experiments from DGCs born during development (Pup) and adulthood (Adult) upon stimulation of the MPP (*n* = 15 pairs, *p* = 0.3, paired *t*-test) or LPP (*n* = 11 pairs, *p* = 0.049). Mean ± SEM is shown on the sides. Recordings were carried out in the presence of 20 μM BMI in slices obtained from mice aged 19–21 wk. Neurons were approximately 18 wk old (P7) and approximately 13 wk old (P42). All plots depict mean ± SEM.

At a negative holding potential (V_Hold_ = −80 mV), EPSCs are mostly mediated by AMPA receptors [42]. Paired comparisons between DGCs born during development and adulthood revealed a small difference in EPSC amplitude evoked by LPP, but not MPP, stimulation (Figure 2G). At V_Hold_ = +50 mV, a slow N-methyl-D-aspartate (NMDA) component became apparent in both neuronal types (Figures 2D and 3A). The AMPA/NMDA ratio was higher for LPP than for MPP stimulation, but similar for both GFP^+^ and RFP^+^ neurons (Figure 3B).

**Figure 3:**
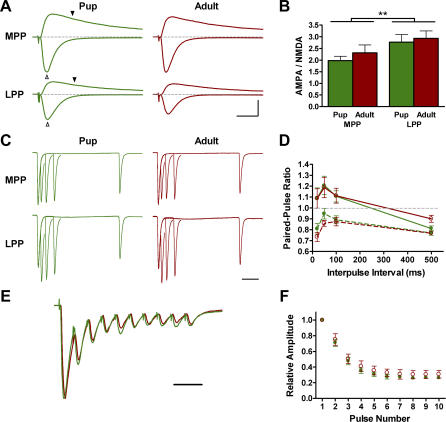
Short-Term Plasticity of Entorhinal Glutamatergic Afferents (A) Average EPSCs recorded at V_Hold_ = −80 mV (downward deflections) and +50 mV (upward deflections) from pup and adult DGCs (*n* = 8 to 11) upon stimulation of MPP or LPP. Dashed line indicates zero level. Arrowheads denote time points for quantification of AMPA (open triangles) and NMDA (filled triangles) currents shown in (B). Criteria for AMPA/NMDA quantification are detailed in the Materials and Methods section. Scale bars indicate 20 ms, 100 pA. (B) AMPA/NMDA ratio from pup and adult DGCs (*n* = 9 to 13) in response to MPP or LPP stimulation. Two-way ANOVA revealed a significant effect of MPP versus LPP (*p* = 0.006), but no significant effect of pup versus adult (*p* = 0.63). (C) Averages of EPSCs in response to paired-pulse stimulation of the MPP or LPP delivered at increasing interpulse intervals (20, 50, 100, and 500 ms). Traces are averages of 7–14 cells aligned and normalized to the first EPSC. Stimulation artifacts and late decay phases of the second EPSC were removed for clarity. Scale bar indicates 100 ms. (D) Paired-pulse ratio as a function of interpulse interval for the experiments shown in (C). Two-way ANOVAs revealed a significant effect of interpulse interval for MPP (dashed lines, *p* < 0.0001) and LPP (solid lines, *p* < 0.0001), but no significant effect of pup (green lines, solid circles) versus adult (red lines, open circles) for either MPP (*p* = 0.073) or LPP (*p* = 0.72) stimulation (*n* = 9 to 14) (E) Example of EPSCs from a pup (green) and an adult DGC (red) in response to MPP stimulation (ten pulses, 50 Hz) Traces are normalized to the first EPSC amplitude. Scale bar indicates 40 ms. (F) Relative EPSC amplitudes measured in response to 50-Hz stimulation evoked as shown in (E). No difference was found between pup and adult responses (two-way ANOVA, *p* = 0.49, *n* = 10 pups [solid circles], *n* = 4 adults [open circles]). All recordings were carried out in the presence of 20-μm BMI. Neurons were approximately 18 wk old (pup) and approximately 13 wk old (adult). All plots depict mean ± SEM.

Certain features of synaptic transmission, including short-term plasticity, can be determined by the phenotype of postsynaptic target cells [43–45]. We therefore reasoned that subtle differences between DGCs generated during development and adulthood might emerge upon induction of short-term plasticity of glutamatergic inputs. The effects of paired-pulse stimulation are known to depend on the inter-pulse interval [41]. MPP stimulation elicited paired-pulse depression with similar extent and time dependence in both neuronal types (Figure 3C and 3D). Likewise, paired stimuli delivered to the LPP elicited a facilitation that displayed a similar time dependence and extent in all neurons.

Fine differences in synaptic physiology might be revealed under conditions of a high demand of transmitter release, such as high-frequency stimulation. MPP stimuli delivered at 50 Hz elicited a significant depression of the EPSC amplitude in both pup and adult-born DGCs (Figure 3E and 3F). No significant differences were observed in the extent of synaptic depression between the two cell types. Taken together, our data demonstrate that mature DGCs generated in the developing and adult hippocampus receive excitatory afferents from the perforant path with similar functional characteristics.

### Convergence of GABAergic Connectivity onto Neurons Generated in the Developing and Adult DG: Evoked Responses

Given the striking similarity of glutamatergic inputs onto DGCs born during development and adulthood, it becomes critical to analyze their GABAergic inputs. A distinct GABAergic connectivity would suggest a unique functionality of adult-born neurons, whereas a similar connectivity would strengthen the functional convergence observed for excitatory afferents. DGCs are innervated by different types of GABAergic interneurons: interneurons localized in the granule cell layer (GCL) project mostly onto the perisomatic compartment, whereas interneurons of the molecular layer (ML) impinge onto the dendritic domains [36]. Extracellular stimulation of the GCL in the presence of 4 mM kynurenic acid (kyn; a glutamate receptor antagonist) elicited monosynaptic inhibitory postsynaptic currents (IPSCs) that were fully and reversibly blocked by BMI (Figure 4A). Paired experiments were carried out to compare IPSCs recorded from GFP^+^ neurons born at the pup stage and RFP^+^ neurons born at the adult stage, in the same slices and with the same stimuli. The ages of neurons at the time of recording were approximately 13 wk (P7) and approximately 7 wk (P42) (Figure 2A). IPSCs recorded at V_Hold_ = −80 mV displayed similar amplitude and kinetics in both neuronal types (Figure 4B–4D).

**Figure 4:**
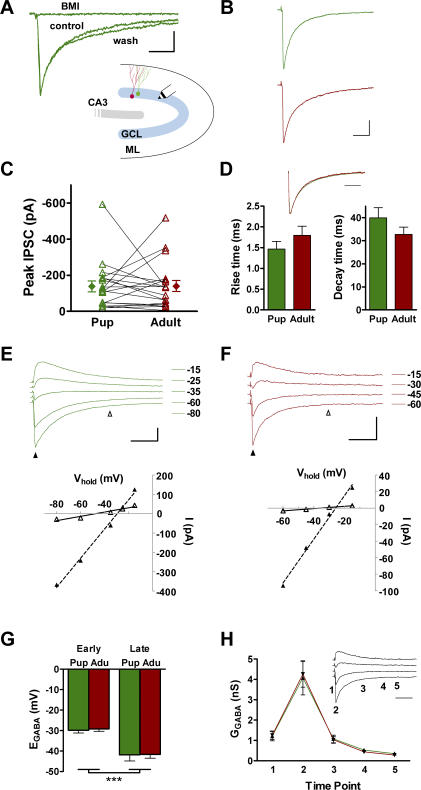
GABAergic Inputs Elicited by GCL Stimulation (A) Example of evoked IPSCs recorded from a pup-born DGC in response to extracellular stimulation of the GCL before (control), during (BMI), and after (wash) application of 20 μM BMI. Scale bars indicate 25 ms, 20 pA. Inset: schematic diagram of the DG showing the position of the bipolar electrode for the stimulation of GCL interneurons. Experiments were carried out with internal solution containing low [Cl^−^]. (B) Typical traces of IPSCs evoked on a pup (green) and adult (red) DGC recorded from the same slice in a paired experiment. Scales indicate 25 ms, 40 pA. (C) Paired analysis of peak IPSC amplitude recorded from a pup versus adult DGCs (*n* = 19 pairs, *p* = 0.97, paired *t*-test). Mean ± SEM are shown on the sides. (D) Kinetics of evoked IPSCs (*n* = 40 adults, *n* = 22 pups; rise time, *p* = 0.31; decay time, *p* = 0.19; unpaired *t*-tests). Inset: scaled average traces of evoked IPSCs. Scale bar indicates 50 ms. (E) and (F) Examples of I-V curves of evoked IPSCs recorded from pup (E) and adult DGCs (F). Top: sample traces of IPSCs recorded at the holding potentials shown on the right (mV). Scale bars indicate 50 ms, 100 pA (E) and 50 pA (F). Bottom: I-V plots measured at time points indicated with arrowheads (early: filled triangles/dashed lines, late: open triangles/solid lines). (G) E_GABA_ measured at early and late time points. Two-way ANOVA (*n* = 11, pup; *n* = 16, adult) revealed a significant effect of early versus late points (*p* < 0.0001), but not of pup versus adult (Adu) neurons (*p* = 0.56). (H) Conductance profile of IPSCs. Each value denotes the slope conductance measured from the I-V curve at the time points indicated on the sample traces (inset). No significant differences were found between pup (green) and adult (red; 11 ≤ n ≤ 16, *p* = 0.62, two-way ANOVA). Scale indicates 50 ms. All experiments were conducted in the presence of kyn (4 mM) in slices obtained from mice aged 13–14 wk. Neurons were approximately 12 wk old (P7) and approximately 7 wk old (P42).

IPSCs elicited by GCL stimulation are typically dominated by a fast perisomatic component, but a slow dendritic current may also be detected [28]. Perisomatic and dendritic IPSCs can be distinguished by their reversal potential (E_GABA_), which is largely determined by the Nernst equilibrium potential for Cl^−^ (E_Cl−_) at the postsynaptic site [46]. In whole-cell recordings, the patch pipette imposes the ionic concentrations at the perisomatic cytoplasm, but not at the distal dendrites, where transporters are more effective at keeping the physiological concentration of intracellular chloride [47–49]. Indeed, a gradient of approximately 12 mV was observed for E_GABA_ between the fast and slow IPSC components, as measured from the current-voltage (I-V) curves (Figure 4E–4G). The difference observed in E_GABA_ under a Cl^−^ load underscores the distinct somatodendritic origin of fast and slow IPSCs [47,49]. No differences were observed between GFP^+^ and RFP^+^ neurons. Notably, the conductance measured from the I-V curves at five time points, which reflects the level of activation of both fast and slow GABAergic inputs, were completely overlapping for GFP^+^ and RFP^+^ neurons, strengthening the remarkable similarity in the afferents from GABAergic interneurons of the GCL (Figure 4H).

Extracellular stimulation of the outer ML elicited GABA_A_-mediated IPSCs with slow kinetics, typical of a dendritic origin [50–52] (Figure 5A and 5B). I-V plots revealed a large E_GABA_ gradient with respect to the somatic E_Cl-_ (∼−35 mV, predicted by the Nernst equation), consistent with the distal nature of IPSCs evoked by ML stimulation (Figure 5C–5E). The maximal GABAergic conductance was somewhat larger in DGCs born during development (Figure 5F), yet no significant differences were observed between DGCs generated in pup and adult mice for any of the parameters assessed.

**Figure 5:**
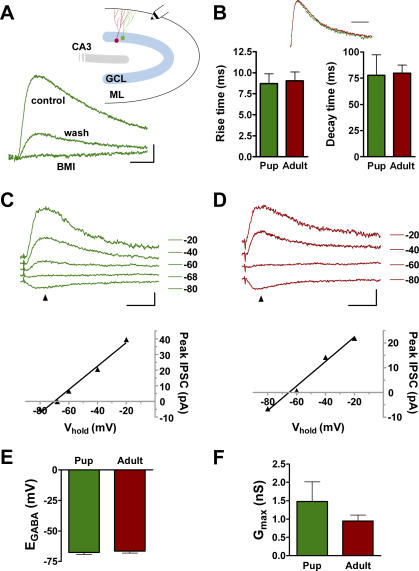
GABAergic Inputs Evoked by ML Stimulation (A) Example of evoked IPSCs recorded from a pup-born DGC in response to extracellular stimulation of the ML before (control), during (BMI), and after (wash) application of 20 μM BMI. Scale bars indicate 25 ms, 20 pA. Inset: schematic diagram of the DG showing the position of the bipolar electrode for the stimulation of ML interneurons. Experiments were carried out with internal solution containing low [Cl^−^]. (B) Kinetics of IPSCs evoked on pup versus adult DGCs (pup, *n* = 6; adult, *n* = 14; rise time, *p* = 0.85; decay time, *p* = 0.90, paired *t*-tests). Inset: scaled averages of IPSCs (pup, green; adult, red). Scale bar indicates 50 ms. (C) and (D) Examples of I-V curves of evoked IPSCs recorded from pup (C) or adult (D) DGCs. Top: sample traces of IPSCs recorded at the holding potentials shown on the right (mV). Scale bar indicates 50 ms, 10 pA. Bottom: I-V plots measured at the time indicated by the arrowheads. (E) E_GABA_ of IPSCs (pup, *n* = 7; adult, *n* = 11; *p* = 0.67, *t*-test). (F) Slope conductance measured at the IPSC peak (*n,* same as in [E]; *p* = 0.23; *t*-test). All experiments conducted in the presence of kyn (4 mM) in neurons aged 12 wk (P7) and 7 wk (P42).

### Convergence of GABAergic Connectivity: Spontaneous Synaptic Activity

GABAergic interneurons are widely distributed within the DG [36]. Therefore, although extracellular stimulation of the GCL and ML can activate numerous GABAergic terminals impinging onto the recorded neuron, most inputs are likely to be beyond the range of the bipolar electrode. To obtain a thorough characterization of GABAergic inputs of DGCs, we have also recorded their spontaneous synaptic activity. Spontaneous IPSCs (sIPSCs) were recorded from GFP^+^ and RFP^+^ DGCs at V_Hold_ = −80 mV under symmetrical Cl^−^ (Figure 6A and 6B). Under these recording conditions, sIPSCs were dominated by fast-rising/fast-decaying currents, typical of perisomatic synapses [47,51,53] (Figure 6C–6E). The frequency and amplitude of detected postsynaptic events were similar for both neuronal types (Figure 6F and 6G). Spontaneous IPSCs recorded from GFP^+^ and RFP^+^ neurons also exhibited similar kinetics, and their scaled average traces were indistinguishable (Figure 6H). Spontaneous events with slow kinetics were also observed, although at a much lower frequency (unpublished data). Slow sIPSCs arise from dendritic compartments, where the lower concentration of intracellular Cl^−^ results in a hyperpolarized E_GABA_ relative to that in the soma [47–49] (see Figures 4 and 5). Consequently, the detection of dendritic sIPSCs was enhanced by carrying out recordings of spontaneous synaptic activity at V_Hold_ = 0 mV, the predicted E_GABA_ for perisomatic sIPSCs under symmetrical Cl^−^. As expected, only slow outward sIPSC were detected under these conditions (Figure 7A and 7B). A wide range of kinetics was observed, presumably reflecting the varying electrotonic distances of the synapses where events originate [53,54]. The frequency, amplitude, and kinetics of dendritic sIPSCs were similar for GFP^+^ and RFP^+^ neurons, highlighting a remarkable similarity in their GABAergic afferent connectivity (Figure 7C–7H).

**Figure 6:**
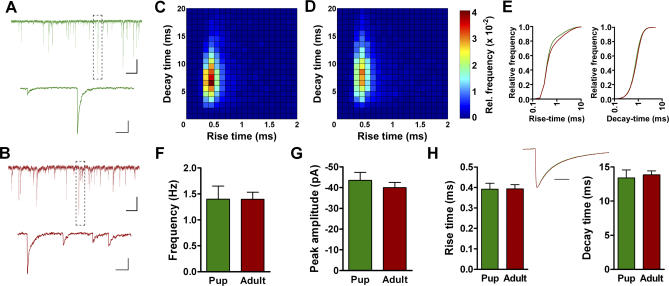
Fast-Perisomatic sIPSCs (A) and (B) Example of traces of inward sIPSCs recorded from a pup (A) and adult (B) DGC. Dashed box on each top trace denotes expanded segment on the bottom. Scale bars indicate 1 s/50 ms (top/bottom), 40 pA. (C) and (D) Two-dimensional histograms of rise and decay time of individual sIPSCs recorded from pup ([C] *n* = 5,871 events) and adult DGCs ([D] *n* = 8,183 events). Color scale indicates the relative frequency for each bin (square areas in the graph). (E) Cumulative histograms of rise and decay time of all sIPSCs recorded from pup (green) and adult (red) DGCs. Data are the same as shown in (C) and (D). (F) Frequency of sIPSCs (pup, *n* = 12 neurons; adult, *n* = 15; *p* = 0.99; *t*-test). (G) Peak amplitude of sIPSCs (*n*, same as in [F]; *p* = 0.44). (H) Kinetics of sIPSCs. Inset: scaled averages of sIPSCs (pup, green; adult, red). Scale bar indicates 10 ms. All experiments conducted in the presence of kyn at V_hold_ = −80 mV with an internal solution containing high [Cl^−^]. (*n*, same as in [F]; rise time, *p* = 0.96; decay time, *p* = 0.72).

**Figure 7:**
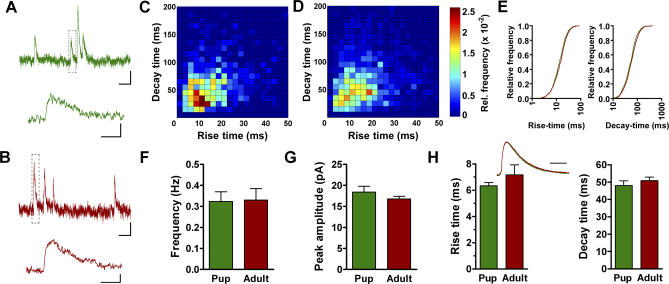
Slow-Dendritic sIPSCs (A) and (B) Example of traces of outward sIPSCs recorded from a pup (A) and adult (B) DGC. Dashed box on each top trace denotes expanded segment on the bottom. Scales indicate 0.5 s/50 ms (top/bottom), 10 pA. (C) and (D) Two-dimensional histograms of rise and decay time of individual sIPSCs recorded from pup ([C] *n* = 695 events) and adult DGCs ([D] *n* = 1,160 events). Color scale indicates the relative frequency for each bin. (E) Cumulative histograms of rise and decay time of all sIPSCs recorded from pup (green) and adult DGCs (red). Same data as shown in (C) and (D). (F) Frequency of sIPSCs (pup, *n* = 10 neurons; adult, *n* = 16; *p* = 0.94; *t*-test). (G) Peak amplitude of sIPSCs (pup, *n* = 10; adult, *n* = 14; *p* = 0.44). (H) Kinetics of sIPSCs. Inset: scaled averages of sIPSCs (pup, green; adult, red). Scale bar indicates 50 ms. All experiments conducted in the presence of kyn at V_hold_ = 0 mV with an internal solution containing high [Cl^−^]. (*n*, same as in [F]; rise time, *p* = 0.37; decay time, *p* = 0.41).

### Firing Behavior Elicited by Excitatory Inputs

Integration of synaptic inputs shapes the firing behavior, which ultimately determines the impact of a neuron in the network. To establish whether adult-born neurons are able to spike in response to an excitatory drive, action currents were recorded under the cell-attached configuration, a form of tight-seal extracellular recording [55]. This non-invasive method provides a high sensitivity for detecting extracellular action potentials while preserving the cytoplasmic environment intact [56]. Indeed, stimulation of the MPP in the presence of BMI (20 μM) elicited action currents whose probability of occurrence increased as a function of the stimulus strength (Figure 8A). Spiking probability of neurons born in the developing and adult hippocampus were compared by simultaneous dual recordings of action currents in response to MPP stimulation (Figure 8B and 8C). Interestingly, whereas differences in spiking probability were observed for each pair of neurons at a given stimulus intensity, no trend was observed in favor of either neuronal age (Figure 8D). These observations demonstrate that DGCs generated in pup and adult mice can integrate excitatory inputs in a similar fashion.

**Figure 8:**
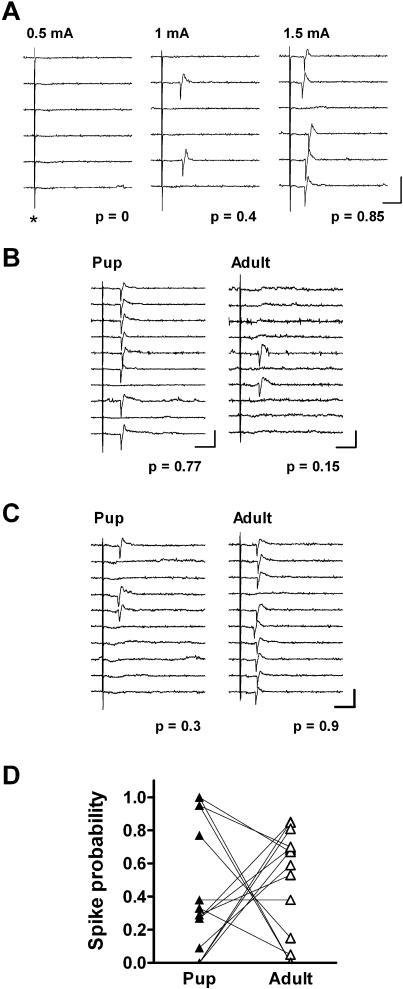
Firing Behavior Elicited by Excitatory Inputs (A) Action currents in cell-attached configuration recorded from an adult-born DGC in response to MPP stimulation at increasing stimulus strengths (0.5–1.5 mA, 50 μs). Six representative epochs are shown. Spiking probability (p) is shown below the traces. The asterisk (*) marks the stimulation artifact. Scale indicates 10 ms, 50 pA. (B) Sample experiment of simultaneous cell-attached recordings of DGCs born in pup and adult brain in response to MPP stimulation (0.4 mA, 50 μs). Action currents indicate a higher spiking probability in the pup DGC. Scales indicate 10 ms, 50 pA (left) and 20 pA (right). (C) Sample experiment in which the spiking probability is higher in the adult-born DGC (1.5 mA, 50 μs). Scale indicates 10 ms, 30 pA. (D) Firing behavior of DGCs born in pup and adult brain during simultaneous paired experiments. No significant difference was found (*n* = 14 pairs, *p* = 0.8, Wilcoxon signed rank test). All recordings were carried out in the presence of BMI (20 μM). In this set of experiments, adult-born neurons were retrovirally labeled with GFP, whereas unlabeled DGCs of the middle third of the GCL were considered postnatally born (see Materials and Methods). Repetitive (>15 episodes) slow frequency stimulation was used to measure the spiking probability for each neuron at the given stimulus.

## Discussion

To better understand the physiological relevance of adult hippocampal neurogenesis, we compared the functional properties of DGCs generated in the developing and adult hippocampus. Double retroviral labeling was used to unequivocally identify neurons generated in these different stages and to study their major excitatory and inhibitory inputs. The striking functional similarity observed here is particularly surprising in light of the numerous parameters quantitatively assessed. For glutamatergic afferents, we have measured the strength, kinetics, AMPA/NMDA ratio, and short-term plasticity of responses evoked by both MPP and LPP stimulation. For GABAergic afferents, we have monitored amplitude, conductance, reversal potential, and kinetics of evoked responses elicited by stimulation of the GCL and ML, as well as frequency, amplitude, and kinetics of spontaneous postsynaptic responses originating both at the soma and the dendrites. Moreover, all neurons displayed similar intrinsic membrane properties, although a smaller membrane capacitance was observed in adult-born DGCs (Table 1). The overall coincidence of inputs for both GABA and glutamate strongly suggests that the connectivity is conserved and, consequently, the excitation–inhibition balance is maintained. In addition, entorhinal excitatory afferents are integrated in a similar fashion, since action potentials in adult-born DGCs display an overall behavior that is equivalent to that of neurons born in the perinatal brain. The impact of the functional convergence observed here is discussed below.

**Table 1:**
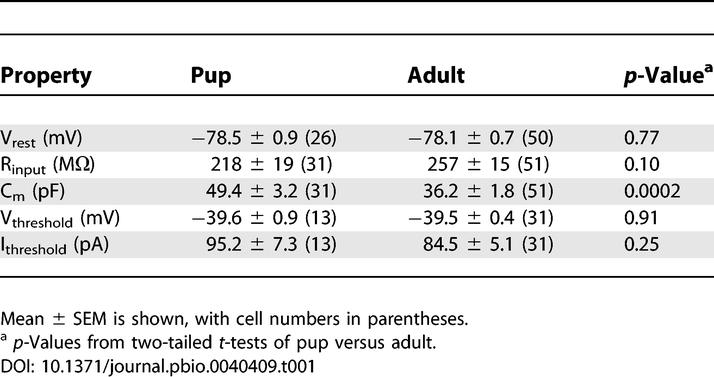
Intrinsic Properties of DGCs Born in the Early Postnatal and Adult DG

### Implications for Hippocampal Function

A thorough understanding of the biological significance of adult neurogenesis in hippocampal function demands complementary approaches: investigating how hippocampal-dependent behaviors are affected by changes in adult neurogenesis (a top-down strategy) [9,11–15,57], and determining the functional impact of individual neurons that become incorporated into the circuits (a bottom-up strategy used in this study). Our approach would predict different roles for mature neurons generated in the adult DG depending on whether they become a distinct functional population or they remain similar to neurons generated during development. In the former case, adult-born DGCs might contribute with a unique functionality to the existing network such as different synaptic plasticity or connectivity that is absent in developmentally generated neurons. The functional convergence observed in our study is in agreement with the latter case and supports a model in which recruitment of neurons belonging to the same functional population is required in hippocampal processing. Mathematical simulations have proposed selective death of old neurons as a mechanism to improve learning performance of neuronal networks with active neurogenesis [58–60]. This notion assumes that newly generated neurons are recruited to replace old DGCs that undergo apoptotic death. Whether adult neurogenesis results in replacement or in a net addition of DGCs remains controversial and awaits further investigation [61–65]. Continuous addition of DGCs could be detrimental to network function, should no homeostatic mechanism compensate for the increased excitation [66]. In light of our observations that newly incorporated DGCs share a common synaptic drive with the preexistent neurons, putative homeostatic mechanisms might be expressed at the output or network level.

We have focused on neurons that have reached functional maturity, yet the uniqueness of adult-born DGCs might emerge at immature stages. In fact, young DGCs of the adult hippocampus display a low threshold for the induction of long-term potentiation [20–22], and their intrinsic membrane properties and excitation–inhibition balance are very different from those described in this work for fully mature, adult-born neurons [22,28,29,31]. Immature neurons with enhanced plasticity could be relevant to hippocampal function provided they were capable of neurotransmitter release onto a target cell, a question that has not yet been addressed either for immature or for mature adult-born DGCs. A recent hypothesis proposes a critical role for young DGCs with a higher excitability in the association of new memories that occurred within a restricted temporal window [67]. In the olfactory bulb (OB), the other neurogenic region of the adult brain, a recent study has revealed that neurons generated in response to an enriched odor environment survive for a short period (<1 mo) and may participate in memory formation [68]. Since complete maturation within this short period is unlikely, immature neurons of the OB might already participate in odor processing. Our observations demonstrate that DGCs generated in the adult hippocampus become fully integrated into the afferent network and remain functional for at least 4 mo. Such stability in the function and connectivity establishes newly generated neurons as a suitable substrate for information storage.

### Possible Mechanisms of Functional Convergence

Our study does not provide evidence on the identity and specific location of presynaptic neurons or on the effects of other neurotransmitters and neuromodulators not considered here. However, the remarkable functional similarity in afferent connections of GABAergic and glutamatergic nature points to a similar balance between excitation and inhibition in DGCs generated during development and adulthood. The relationships between excitation, inhibition, and excitability can be influenced by different mechanisms of neuronal homeostasis [69]. For instance, neurons can adjust their gain in order to maintain defined levels of activity. Cultured neurons reduce or increase their excitatory drive when exposed to prolonged periods of hyperactivity or deprivation by modulating the number of postsynaptic AMPA receptors, a phenomenon called synaptic scaling [70]. NMDA receptors are co-regulated so that the AMPA/NMDA ratio is maintained in both homeostatic and Hebbian plasticity [71]. In this context, the differences in the AMPA/NMDA ratio between MPP and LPP afferents (Figure 3B) would suggest differential regulatory mechanisms acting at these synapses. Opposite homeostatic effects are also present for afferent inhibition [69]. In light of the functional convergence observed here, it is tempting to speculate that similar homeostatic mechanisms are shared by all DGCs, regardless of their origin.

Whether the ability to support functional convergence in the adult brain is restricted to neurogenic environments or is a widespread phenomenon is relevant to tailoring strategies for neuronal replacement in neurodegeneration and trauma. Methods involving induction of endogenous neuronal production and recruitment and/or stem cell transplantation would both require that newly incorporated neurons be able to establish appropriate connections with afferent and target networks. Neuronal replacement therapies are more likely to become successful if grafted neurons serve a function that is similar to that of neurons affected by pathological conditions [72].

## Materials and Methods

### Viral vectors.

A replication-deficient retroviral vector based on the Moloney murine leukemia virus was used to express enhanced GFP or mRFP1 (RFP) [73] driven by a CAG promoter [39]. Retroviral particles were assembled using three separate plasmids containing the capside (CMV-vsvg), viral proteins (CMV-gag/pol), and transgene (CAG-GFP or CAG-RFP). Plasmids were transfected onto 293T cells using Lipofectamine 2000 (Invitrogen, Carlsbad, California, United States). Virus-containing supernatant was harvested 48 h after transfection and concentrated by two rounds of ultracentrifugation.

### Subjects and stereotaxic surgery.

C57Bl/6J P7 pups were anesthetized by placing them on ice for 4 min. CAG-GFP expressing retrovirus was infused (0.7 μl in 1 min) into the dorsal area of the right DG (coordinates from bregma: antero-posterior = −1.5 mm, lateral = 1.5 mm, ventral = 1.5 mm) using a microcapillary calibrated pipette (Drummond Scientific, Broomall, Pennsylvania, United States). Pups were placed back with their mother after recovery (30–60 min on a warm pad). After weaning (at P21), mice were segregated by sex. At P42–P49, mice were anesthetized (100 μg ketamine/10 μg xylazine in 10 μl saline/g) and a second surgery was carried out to infuse the CAG-RFP retrovirus (0.7 μl in 5 min) into the dorsal aspect of the right DG (coordinates: −2 mm, 1.5 mm, 1.9 mm). Three days before the second surgery and thereafter, mice were housed with a running wheel to increase the efficiency of viral transduction [1,28].

### BrdU treatment.

After weaning (P21), mice received a daily intraperitoneal injection of BrdU 50 mg/kg over 5 d. Four weeks after the last injection, mice were anesthetized (ketamine/xylazine) and perfused intracardially with 4% paraformaldehyde (PFA). Brains were removed and sectioned (40 μm) in a cryostat (Leica, Wetzlar, Germany). One in six sections throughout the hippocampus were studied by immunohistochemistry and confocal microscopy, and all sections containing GFP^+^ neurons were included in the analysis. Neuronal phenotype of GFP^+^ cells was assessed by morphology. The phenotype of GFP^+^/BrdU^+^ cells was corroborated by NeuN labeling.

### Immunofluorescence and confocal microscopy.

Immunostaining for BrdU was done on 40-μm free-floating coronal sections. Double labeling of GFP and RFP was analyzed in 400-μm sections cut with a vibratome and fixed with 4% PFA. Antibodies were applied in TBS with 3% donkey serum and 0.25% Triton X-100. Primary antibodies were: BrdU (rat; 1:200; Boehringer Manheim, Roche, Basel, Switzerland) and GFP (chicken; 1:500; Invitrogen). NeuN (mouse monoclonal; 1:50) was included in all sections to label the GCL and to assess the neuronal phenotype. Corresponding secondary antibodies were used (donkey anti-chicken Cy2, donkey anti-rat Cy3, donkey anti-mouse Cy5; 1:250; Jackson ImmunoResearch, West Grove, Pennsylvania, United States). Images were taken with an optical pinhole of 1 μm using a Zeiss Pascal confocal microscope (Zeiss, Oberkochem, Germany). Only GFP^+^ cells located in the subgranular zone (SGZ) or GCL were included in the analysis. Co-localization was analyzed in single optical planes taken through the entire *z*-axis of each cell.

### Electrophysiology: Whole-cell recordings.

Experiments were carried out in 56 slices from 30 mice injected at P7 and P42 (12 male and 18 female). Experiments on perforant path afferents were carried out 13.6 ± 0.5 wk (range: 12.9 to 14.6) after the second (P42) surgery. Experiments on GABAergic inputs and intrinsic membrane properties were carried out 6.7 ± 0.4 wk (range: 6.0 to 7.6) after the second (P42) surgery. Mice were anesthetized and decapitated. Brains were removed into a chilled solution containing (mM): 110 choline-Cl^−^, 2.5 KCl, 2.0 NaH_2_PO_4_, 25.0 NaHCO_3_, 0.5 CaCl_2_, 7 MgCl_2_, 20 dextrose, 1.3 Na^+^-ascorbate, 0.6 Na^+^-pyruvate, 4 kyn. Right hemisphere slices (400-μm thick) were cut in a vibratome and transferred to a chamber containing artificial cerebrospinal fluid (ACSF; mM): 125.0 NaCl, 2.5 KCl, 2.0 NaH_2_PO_4_, 25.0 NaHCO_3_, 2 CaCl_2_, 1.3 MgCl_2_, 1.3 Na^+^-ascorbate, 3.1 Na^+^-pyruvate, and 10 dextrose. Our stereotaxic injections mostly labeled dorsal DGCs. Experiments on perforant path afferents were carried out in coronal sections to render slices transversal to the dorsal hippocampus (the number of transverse dorsal slices was optimized by including a subtle angle to coronal sectioning). Experiments on GABAergic inputs were performed in horizontal slices. Slices were bubbled with 95% O_2/_ 5% CO_2_ (315 mOsm) and maintained at 30 °C during recovery. Recordings were carried out in ACSF at 23 ± 1 °C using microelectrodes (5–8 MΩ) pulled from borosilicate glass (KG-33, Garner Glass, Claremont, California, United States). ACSF was supplemented with 20 μM BMI for EPSCs recordings and 4 mM kyn for IPSCs recordings. Four internal solutions were used; all of them included (mM) 10 Hepes, 4 Tris-ATP, 0.3 Tris-GTP, 10 phosphocreatine, and Alexa Fluor 488 or 594 (10 μg/ml; Invitrogen), (pH 7.3) and 290 mOsm. In addition, EPSCs were recorded using (mM): 130 Cs-gluconate, 10 tetraethylammonium-Cl, 2 NaCl, 2 MgCl_2_, 0.2 EGTA, 2 QX-222; evoked IPSCs and intrinsic membrane properties were recorded with (mM): 120.0 Kgluconate, 20 KCl, 5 NaCl, 4 MgCl_2_, 0.1 EGTA; for spontaneous IPSC at V_Hold_ = −80 mV we used (mM): 19 Kgluconate, 121 KCl, 5 NaCl, 4 MgCl_2_, 0.1 EGTA; and for spontaneous IPSC at V_Hold_ = 0 mV, we used (mM): 140 CsCl, 5 NaCl, 2 MgCl_2_, 0.1 EGTA. Unless otherwise noted, all chemicals were from Sigma (St. Louis, Missouri, United States).

Whole-cell recordings (Axopatch 200B; Molecular Devices, Sunnyvale, California, United States) were filtered at 2 kHz, digitized (Digidata 1322A, Molecular Devices) and acquired onto a PC using jClamp (SciSoft [http://www.scisoftco.com/jclamp.html)]. Series resistance was typically 15–25 MΩ. Criteria to include cells in the analysis were (1) co-labeling with Alexa Fluor 594 (for GFP^+^ cells) or 488 (for RFP^+^ cells) or visual confirmation of GFP or RFP fluorescence in the pipette tip, and (2) absolute leak current less than 150 pA. Unless otherwise noted, voltage-clamp recordings were performed at V_Hold_ = −80 mV. Extracellular stimulation (50 μs, 0.1 Hz) was done using concentric bipolar electrodes (50-μm diameter; FHC, Bowdoinham, Maine, United States), a Grass S88 stimulator and a stimulus isolation unit (SIU5, Grass Instruments, West Warwick, Rhode Island, USA). In paired experiments, a development-born DGC (GFP^+^) and one neighboring adult-born DGC (RFP^+^) were sequentially recorded while maintaining electrode position, stimulation protocols, and stimulus strength (mostly 10 V, 50 μs) invariable. The order in which GFP^+^ and RFP^+^ cells were recorded was alternated across experiments. For orthodromic stimulation of MPP and LPP afferents, two stimulation electrodes were positioned on the middle and outer third of the ML, respectively, towards the crest of the DG. This resulted in appropriate separation of MPP versus LPP, as evidenced by the paired-pulse ratio and EPSC kinetics (Figures 2E and 2F). For stimulation of GABAergic interneurons, the stimulation electrode was positioned on the GCL or outer third of the ML, as stated in the text. Electrodes were positioned approximately 250 μm away from the recorded cell. When more than one DGC of the same group was recorded for the same paired experiment, average values were used for comparisons.

The localization of the rare P7-born DGCs that continued to divide 2 wk after labeling (Figure 1E) was restricted to the inner half of the GCL, whereas P7 neurons of the outer half did not incorporate BrdU. To exclude those rare neurons from our analysis, only P7-born DGCs of the outer half were selected for electrophysiological recordings.

### Electrophysiology: Cell-attached recordings.

In this set of experiments, we have labeled neurons born at P42 using the CAG-GFP retrovirus, and took unlabeled (GFP^−^) DGCs of the middle third of the GCL as pup born (see Figure 1A and 1B; E. A. Mathews, N. Morgenstern, V. Piatti, S. Jessberger, A. F. Schinder, F. H. Gage, unpublished data). In additional support of this criterion, it has been shown that adult-born neurons largely remain within the inner third of the GCL [28,74]. Recordings were carried out in 11 slices from eight mice, approximately 9 wk after infection. Simultaneous cell-attached recordings were carried out under voltage clamp at 0 mV using pipettes with a high tip resistance (10–14 MΩ). Stimuli were delivered to the middle third of the ML. Stimulus intensity was gradually increased (0.3–3 mA, 50 μs) and spiking probability for each neuron was obtained after 15–30 stimuli repeated at 15-s intervals. For each pair of cells, the single stimulus intensity that rendered the largest difference in spiking probability was selected for statistical analysis (Figure 8D). Only experiments in which spiking was detected in both neurons were considered for analysis. Recordings were discarded if the seal resistance reached values below 8 GΩ. The integrity of the cell-attached patch was further confirmed by the absence of fluorescent dye in the cytoplasm.

### Analysis of electrophysiological data.

Passive properties were calculated from the current response to a 5-mV hyperpolarizing step. Series and input resistances were measured from the peak and steady state values of the negative deflection; membrane capacitance was obtained after measuring the area under the capacitive current. Evoked postsynaptic currents were analyzed from average traces using ad hoc MATLAB (The Mathworks [http://www.mathworks.com/products/matlab/]) routines. For AMPA/NMDA ratios, AMPA was measured as the peak amplitude of the EPSC at V_hold_ = −80 mV. NMDA was measured as the EPSC amplitude at V_hold_ = +50 mV, 33 ms after the stimulus artifact (∼25 ms after the AMPA peak; arrowheads in Figure 3A). At this time point, AMPA currents decay to less than 5% of their peak amplitude, and their contribution to the NMDA component becomes negligible [42]. The overall significance of the comparisons of AMPA/NMDA ratio for each group was not dependent on the time point chosen for measuring the NMDA component (unpublished data).

In paired-pulse recordings, a baseline value was measured prior to the second stimulation artifact and subtracted from the amplitude of the second EPSC. For inter-pulse intervals less than 100 ms, the baseline value was extrapolated from the monoexponential fitting of the first EPSC decay phase. Rise and decay times were calculated from 20% to 80% and 100% to 40% of the peak amplitude, respectively. For individual sIPSC recorded at 0 mV (Figure 7), decay time represents time constant from single-exponential fits of the decay phase. Kinetics of IPSCs evoked on the GCL and ML were measured from inward and outward traces recorded at V_Hold_ = −80 and approximately −40 mV, respectively. For GCL-evoked IPSCs, E_GABA_ and G_GABA_ were calculated from I-V curves constructed at five time points: (1) the rising phase of the IPSC; (2) the peak of the IPSC at V_Hold_ = −80 mV; and at (3) 85 ms, (4) 145 ms, and (5) 185 ms after the stimulation artifact. For ML-evoked IPSCs, E_GABA_ and G_GABA_ were calculated from I-V curves constructed at the time of the outward IPSC peak. In some experiments, the current amplitude at V_Hold_ = −80 mV deviated from linearity and was not considered for the I-V fitting. All sample traces of evoked responses are averages of more than ten sweeps.

Spontaneous IPSC were sampled at 10 KHz with Axoscope 9 (Molecular Devices), digitally filtered at 1 KHz, and detected offline with Mini Analysis software (Synaptosoft [http://www.synaptosoft.com/MiniAnalysis/]). Amplitude and area thresholds for detection were 10 pA and 50 pA.ms, and the average standard deviation of baseline noise was 2 pA. All events were individually validated, and artifacts were discarded by visual inspection. For analysis of sIPSC peak amplitude (Figures 6G and 7G), a mean value was obtained for each DGC. Individual sIPSCs were then exported to MATLAB, where kinetics were analyzed and two-dimensional histograms were obtained using routines developed in our laboratory. Kinetics of sIPSCs (Figures 6H and 7H) were analyzed from the scaled average sIPSC from each cell. Events with multiple peaks were discarded from the analysis. For the construction of sIPSC scaled averages, traces were aligned at 10% of the rising phase. All statistical tests used two-tailed analysis.

## Abbreviations

ANOVA: analysis of variance
BMI: bicuculline methiodide
BrdU: bromodeoxyuridine
DG: dentate gyrus
DGC: dentate granule cell
EC: entorhinal cortex
EPSC: excitatory postsynaptic current
GCL: granule cell layer
GFP: green fluorescence protein
IPSC: inhibitory postsynaptic current
I-V: current-voltage
kyn: kynurenic acid
LPP: lateral perforant path
ML: molecular layer
MPP: medial perforant path
NMDA: N-methyl-D-aspartate
NPC: neural progenitor cell
P: postnatal day
RFP: red fluorescent protein
SEM: standard error of the mean
sIPSC: spontaneous inhibitory postsynaptic current
